# Discovery of mammalian genes that participate in virus infection

**DOI:** 10.1186/1471-2121-5-41

**Published:** 2004-11-02

**Authors:** Edward L Organ, Jinsong Sheng, H Earl Ruley, Donald H Rubin

**Affiliations:** 1 Research Medicine, VA Tennessee Valley Healthcare System, Nashville, TN, USA; 2 Department of Medicine, Division of Infectious Diseases, Vanderbilt University, Nashville, TN, USA; 3 Department of Microbiology and Immunology, Vanderbilt University, Nashville, TN, USA

## Abstract

**Background:**

Viruses are obligate intracellular parasites that rely upon the host cell for different steps in their life cycles. The characterization of cellular genes required for virus infection and/or cell killing will be essential for understanding viral life cycles, and may provide cellular targets for new antiviral therapies.

**Results:**

Candidate genes required for lytic reovirus infection were identified by tagged sequence mutagenesis, a process that permits rapid identification of genes disrupted by gene entrapment. One hundred fifty-one reovirus resistant clones were selected from cell libraries containing 2 × 10^5 ^independently disrupted genes, of which 111 contained mutations in previously characterized genes and functionally anonymous transcription units. Collectively, the genes associated with reovirus resistance differed from genes targeted by random gene entrapment in that known mutational hot spots were under represented, and a number of mutations appeared to cluster around specific cellular processes, including: IGF-II expression/signalling, vesicular transport/cytoskeletal trafficking and apoptosis. Notably, several of the genes have been directly implicated in the replication of reovirus and other viruses at different steps in the viral lifecycle.

**Conclusions:**

Tagged sequence mutagenesis provides a rapid, genome-wide strategy to identify candidate cellular genes required for virus infection. The candidate genes provide a starting point for mechanistic studies of cellular processes that participate in the virus lifecycle and may provide targets for novel anti-viral therapies.

## Background

Cellular genes are likely to participate in all phases of viral life cycles including attachment to cellular receptors, internalization, disassembly, translation of mRNA, assembly and egress from the cells [1]. The susceptibility to virus infection varies greatly among different cell types, and virus-resistant cells frequently emerge post-infection [2-4]. This suggests that genetic determinants can influence host cell contributions to the virus life cycle. Despite examples of mammalian genes that influence virus infection, the identification of such genes has been hampered by the lack of practical methods for genetic analysis in cultured cells. In the present study, we tested whether tagged sequence mutagenesis – a gene entrapment strategy widely used to mutate genes in mouse embryonic stem cells [5-10] could be used to identify candidate cellular genes required for lytic infection by reovirus, a small cytolytic RNA virus that replicates in the cytoplasm. The mammalian reoviruses serve as useful models for virus-host cell interaction due to their capacity to replicate preferentially in proliferating and undifferentiated cells [3].

Gene traps are efficient mutagens as assessed by studies in mice of mutations induced originally in embryonic stem cells. In somatic cells, the approach assumes that loss-of-function mutations induced by gene entrapment may confer reovirus resistance as a result of gene dosage effects (e.g. haploinsufficiency), pre-existing heterozygosity or loss of heterozygosity. Following infection with the U3NeoSV1 retrovirus gene trap shuttle vector, libraries of mutagenized rat intestinal epithelial (RIE)-1 cell clones were isolated in which each clone contained a single gene disrupted by provirus integration [6]. The entrapment libraries were infected with reovirus type 1, and virus-resistant clones were selected under conditions that also selected against the emergence of persistently infected cells (PI) that may express virus resistance in the absence of cellular mutations [4]. Genes disrupted in a total of 151 reovirus resistant cells were identified by sequencing regions of genomic DNA adjacent to the entrapment vector [6]; of these, 111 contained mutations in previously characterized genes and anonymous transcription units.

Reovirus-resistant clones were selected at higher frequencies from entrapment libraries than from non-mutagenized cells, suggesting that reovirus-resistant phenotypes were induced by gene trap mutagenesis. However in any genetic screen, clones with the selected phenotype may arise from spontaneous mutations, and consequently, additional experiments are required to demonstrate that individual genes disrupted by gene entrapment actually contribute to the reovirus-resistant phenotype. For example, a mutation in *Ctcf *mutation, a transcriptional repressor of insulin growth factor II (IGF-II), was one of 4 mutations associated with reovirus resistance that affected IGF-II expression and/or signalling. Subsequent experiments demonstrated that enforced IGF-II expression is sufficient to confer high levels of reovirus resistance [4]. In short, genes collectively identified by tagged sequence mutagenesis in a panel of reovirus resistant clones provide candidates for mechanistic studies of cellular processes that participate in the virus lifecycle. Since the disrupted genes do not adversely affect cell survival, drugs that inhibit proteins encoded by the genes are not expected to be overtly toxic to cells. Hence, the candidate genes may also include targets for novel anti-viral therapies.

## Results

### Tagged sequence mutagenesis and selection of reovirus resistant clones

Twenty libraries of mutagenized RIE-1 cells, each representing approximately 10^4 ^independent gene trap events, were isolated following infection with the U3NeoSV1 gene trap retrovirus. U3NeoSV1 contains coding sequences for a neomycin resistance gene in the U3 region of the viral long terminal repeat (LTR). Selection for neomycin resistance generates clones with proviruses inserted within actively transcribed genes. Cells pooled from each entrapment library were separately infected with Type 1 reovirus at a multiplicity of infection of 35, and reovirus-resistant clones were selected in serum-free media to suppress the emergence of persistently infected (PI) cells (4). A total of 151 reovirus-resistant clones were isolated – approximately 1 mutant per 10^3 ^gene trap clones or 1 mutant per 10^7 ^reovirus infected cells. For comparison, the frequency of recovering resistant clones from RIE-1 cells not mutagenized by gene entrapment was less than 10^-8^. This suggests that reovirus-resistant phenotypes were induced by gene trap mutagenesis.

**Figure 1:**
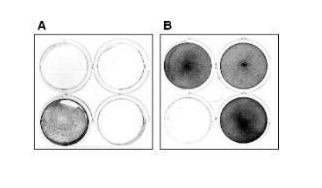
**Characterization of phenotypic properties of cloned RIE-1 cells resistant to reovirus type 1 infection **(A) Cells were stained for reovirus antigen as previously described [3]. Only the PI cells contain reovirus antigen as detected by immunohistochemistry (dark wells). Upper wells contain cloned mutant RIE-1 cells from two sets of RIE-1 mutant cell lines selected for reovirus resistance. The lower wells contain PI RIE-1 (left) and uninfected wild type RIE-1 (right). (B) Reovirus susceptible L-cell monolayers, maintained in 1 ml of completed medium, were used to detect the presence of virus in a 100 μl lysate obtained of mutant cells (upper two wells), PI RIE-1 cells (lower left) or uninfected parental RIE-1 cells (lower right). Note, that only L-cell monolayers exposed to a lysate from PI RIE-1 cells lysed within one week of exposure (gentian violet stain).

**Figure 2:**
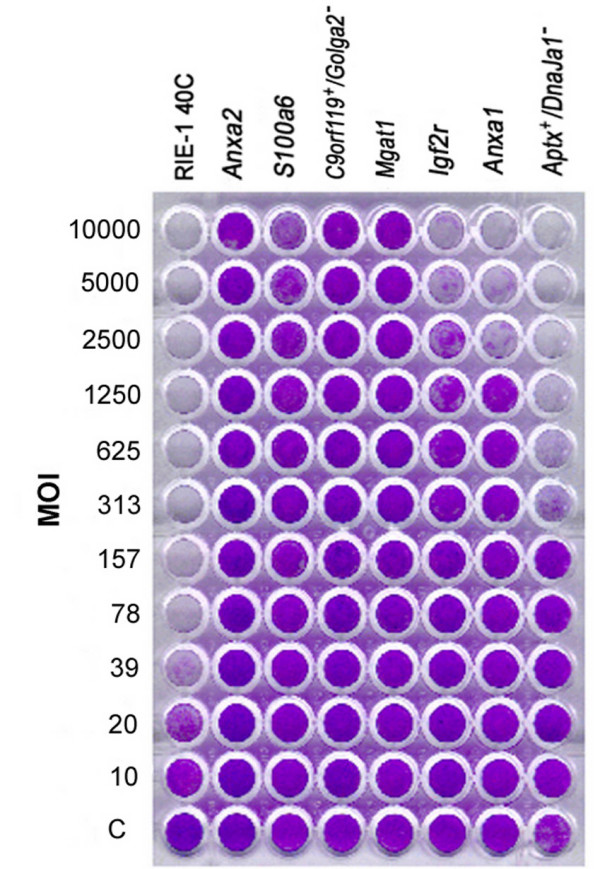
**RIE-1 mutant cells resist lytic infection by reovirus **The columns contain an unselected RIE-1 cell library, RIE-1 40C, and representative reovirus resistant mutant cell clones. Serial two-fold dilutions of reovirus were made with the highest titer in the upper row, MOI = 1 × 10^4^. Resistance to reovirus type 1 infection was observed in the mutant cells 3 to 7 days post-infection. The bottom row of cells, denoted by "C", were not infected to serve as controls for cell viability and proliferation. Cells were stained with gentian violet four days post-infection. A clear well indicates cell death following virus infection.

Reovirus-resistant cells selected in serum-free media did not express viral antigens (Figure 1) and did not produce infectious virus as assessed by plaque assay (E.L. Organ, unpublished results). Most clones were resistant to infection by high titre reovirus and were further analyzed (Figure 2). While reovirus resistance did not initially result from the establishment of a persistent infection, many clones became persistently infected upon subsequent passages, presumably because mutant cells that display virus resistance are susceptible to the establishment of a PI state [2] from residual virus used in selection.

### Identification of genes disrupted in reovirus-resistant clones

The U3NeoSV1 gene trap vector contains a plasmid origin of replication and ampicillin resistance gene; thus, regions of genomic DNA adjacent to the targeting vector were readily cloned by plasmid rescue and sequenced [6]. The flanking sequences were compared to the nucleic acid databases to identify candidate cellular genes that confer resistance to lytic infection by reovirus when altered by gene entrapment. Altogether, the 151 cloned flanking sequences matched 111 annotated gene and transcription units in the public DNA sequence databases [non-redundant (nr), high-throughput genomic sequences (htgs), and human, mouse, and rat genome sequences [6]. 40 flanking sequences were uninformative because they matched repetitive elements or regions of genomic DNA not associated with any annotated transcription unit.

**Figure 3:**
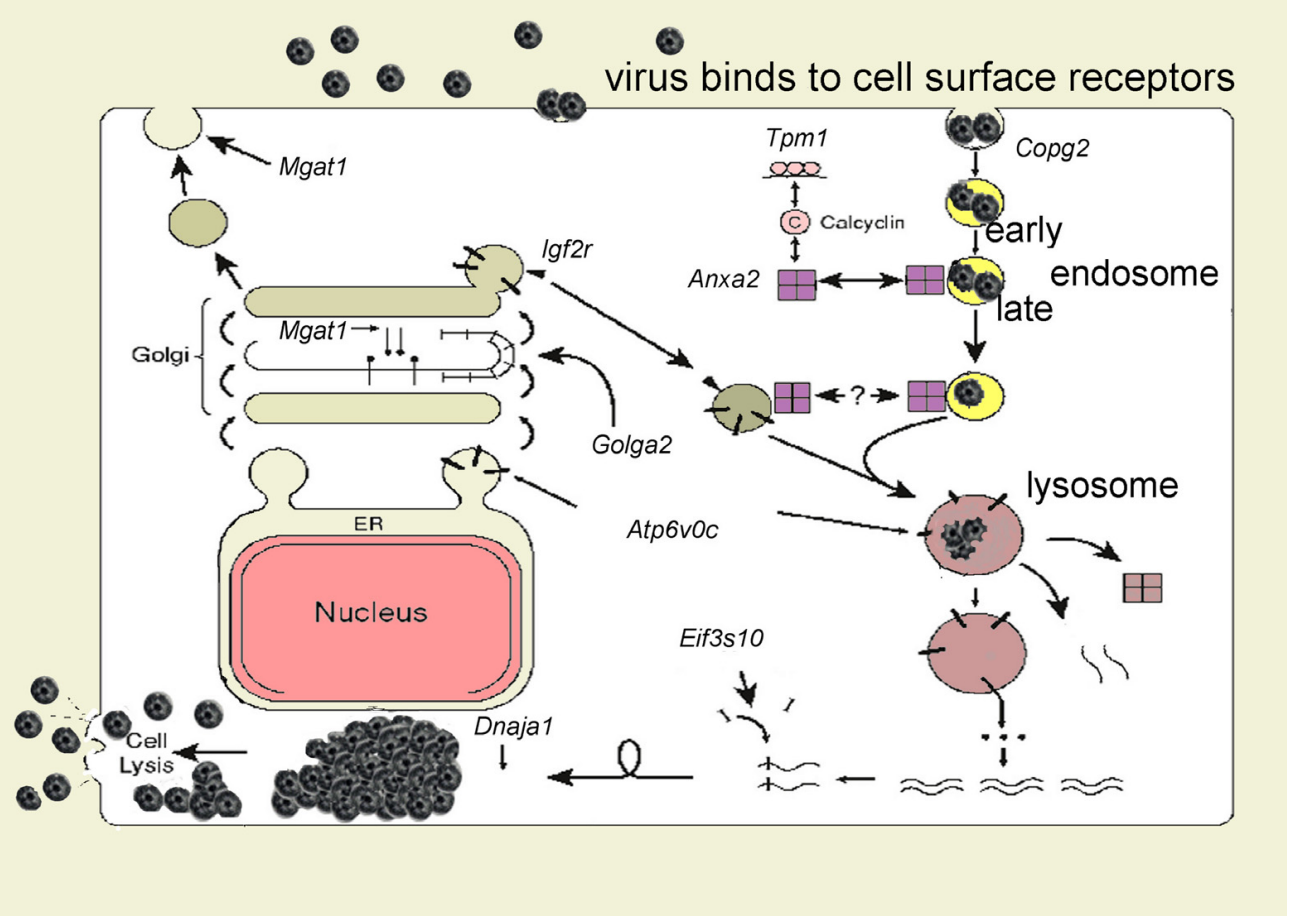
**A model of the life cycle of reovirus: proposed checkpoints based on function of the cellular genes identified by the insertional mutagenesis **The virus life cycle begins (top, clockwise) with virus binding to cell surface receptor and being endocytosed into early endosomes. These endosomes then associate with annexin-II (*Anxa2*) [62] and fuse with annexin-II-associated vesicles containing newly synthesized lysosomal enzymes migrating from the Golgi [63], which further fuse with the lysosome. The vacuolar H^+^-ATPase (*Atp6v0c*) acidifies the lysosome, allowing acid-dependent proteases to digest the outer coat from the virus particles and activate them [64]. These activated particles then pass through the lysosomal membrane and begin transcription of mRNA. The Golgi protein gm 130 (*Golga2*) is believed to mediate the docking of vesicles as they carry their newly synthesized cargo through the Golgi stack [65, 66]. *N*-acetylglucosaminyl transferase I (*Mgat1*) initiates the glycosylation of cell surface proteins (receptors?) and may play a major role, through kinship recognition, in helping maintain the correct assortment of lysosomal enzymes [67-71]. The *Igf2r *shuttles enzymes bound for the lysosome from the Golgi [72] and transfer *Igf2 *to the lysosome. While the roles of calcyclin and the α-tropomyosin (*Tpm1*) are still unclear, they specifically bind each other, and calcyclin is known to bind *Anxa2 *[16, 20]. Thus, they may be involved in endosome fusion. *Eif3s10 *specifically binds the virus message to begin its preferential translation. The *DnaJa1 *protein may facilitate the proper folding of virus proteins with its chaperone function [73]. However, *DnaJa1 *protein and *Eif3 *may play additional roles in virus trafficking or apoptosis, respectively. Eventually, morphogenesis is complete when crystalline-like arrays of new virions form, cell lysis occurs, and virus is released. Many of the cellular proteins encoded by mutated genes have direct or indirect roles in trafficking of endosomes or lysosomal fusion and thus may play roles in the early disassembly or delivery of transcriptionally active virions to the appropriate cell location.

The Supplementary Table [see Additional File 1] lists genes disrupted in reovirus resistant clones for which some functional information is available. Many of these genes encode proteins that are known to physically interact. Genes associated with particular metabolic or signalling pathways are shown in Table 1. These include gene products that could play potential roles in all aspects of virus replication: entry, disassembly, transcription, translation, and reassembly (Table 1, Figure 3, Supplementary Table [see Additional File 1]). Eleven genes encoding calcyclin, insulin growth factor binding protein 5 protease (prss11), type C-like lectin protein (*Clr*)-*f *and -*C*, *Dnaja1-/Aprataxin*+ (*Aprx*), GATA binding protein 4 (*Gata4*), Bcl2 like-1 (*Bcl2l1*); and chromosome 10 open reading frame 3 (*Chr10orf3*) *and myoferlin*, fer-1 like protein 3 *(Fer1l3)*, S100a6 (encoding calcyclin), and two functionally anonymous cDNAs were independently mutated in separate cell libraries (Supplementary Table [see Additional File 1]). The proviruses in these independent of mutant clones were located within 7 to over 1500 nucleotides of each other (data not shown).

**Table 1:** Classification of trapped genes according to function Trapped genes are listed by the official HUGO Gene Nomenclature Committee names, when available. Functional placement of genes or their products are determined by literature assignments. Some genes perform more than one cellular role, and are classified arbitrarily and others have undefined roles.

**Transcription**	**Cytoskeletal-Related**	**Membrane**	**Signalling**	**Vesicle/Trafficking**
*Brd2*	*Anx3*	*Abca4*	*E2ip2*	*Anxa1*
*Brd3*	*Cald1*	*Celsr2*	*Fkbp8*	*Anxa2*
*Ctcf*	*Calm2*	*Csmd2*	*Fusip1*	*Atp6v0c*
*E2f2*	*Kif13b*	*Erbb2ip*	*Gata4*	*Copg2*
*Gtf2e1*	*Mapt*	*OL16*	*Grb2*	*Golga2*
*Hnrpl*	*Ppm1a*	*Pgy1*	*Jak1*	*Hm13*
*Hoxc13*	*Rps18*	*Rab13*	*Madh7*	*Igf2r*
*Hp1-bp74*	*Stmn1*	*Serp1*	*Map3k7ip1*	*Psa*
*Id3*	*Tpm1*		*Pde4b*	*Rabl3*
*Zfp7*			*Rraga*	*Rin2*
*Znf207*			*Ryk*	*S100a6*

**Translation**	**Apoptosis**	**Metabolism**	**Chaperonin**	**Ubiquination/Proteosome**

*Cstf2*	*Bcl2l*	*Gas5*	*Dnaja1*	*Psma7*
*Eif3s10*	*IkBζ*	*Lipc*		*Ube1c*
*Srp19*	*Mical2*	*Mgat1*		
	*Rfp2*	*Pts*		

***Unassigned***				

*Aptx*	*Hspc135*	*Ocil*	*Wdr5*	
*Clr-f*	*Klhl6*	*Ror1*		
*Dlx2*	*Mox2r*	*Scmh1*		
*Dre1*	*Numb*	*Trim52*		

While the presence of multiple, independent mutations in specific genes provides indirect evidence for their involvement in the reovirus lifecycle, the genes could also represent hot spots for gene entrapment. The U3NeoSV1 vector preferentially targets genes with 5' exons that can splice in-frame with a cryptic splice site in the *Neo *gene to produce enzymatically active Neo fusion proteins. As a consequence, mutagenesis by U3NeoSV1 is actually quite biased, such that of 400 mutations characterized in ES cells, one-third involved genes disrupted multiple times, including *Pecam*1 which was targeted 9 times [11]. However, none of the multiply targeted genes associated with reovirus resistance involved previously observed entrapment hotspots. Conversely, over 10% of the mutations identified in ES cells involved genes for RNA binding proteins, a preference not observed among genes collectively associated with reovirus resistance. Only *Madh*7 and *Gas5*, each represented once among the reovirus-resistant clones, were disrupted by U3NeoSV1 in ES cells. Both genes are commonly targeted by other retroviral gene trap vectors and thus probably represent hot spots for gene entrapment [5,8].

### Potential involvement of disrupted genes in virus replication

The genes associated with reovirus resistance can be grouped according to their presumed role in virus entry, disassembly, translation, and maturation. Reovirus enters the host via an endocytic pathway that requires acidification and proteolysis to remove the viral outer capsid. The presumptive roles of several candidate genes would be anticipated to affect virus replication by interfering with virus disassembly. For example the mannose-6-phosphate receptor/insulin-growth factor-2 receptor (*Igf2r*) transports cathepsins to the lysosome [12] and acidification of the lysosome is dependent upon the vacuolar H^+^-ATPase (*Atp6v0c*) [13]. NH_4_Cl is a weak base that interferes with the function of two of the tagged genes, the *Igf2r *and the *Atp6v0c*, and blocks the disassembly of reovirus and several other viruses that enter cells via the endocytic pathway. Moreover, specific inhibitors of the vacuolar H^+^-ATPase gene product have been used to block the infectivity of reovirus and influenza A virus [13,14]. Four mutations in three different genes [*Igf2r*, *Prss11*, a protease associated with insulin binding protein 5, and *Ctcf *a transcriptional repressor of IGF-II] are predicted to affect IGF-II expression and/or signalling. Cells containing the *Ctcf *mutation were subsequently found to express elevated levels IGF-II, while enforced IGF-II expression was sufficient to confer high levels of reovirus resistance. The resistance was caused, at least in part, by a block in virus disassembly [4]. Similarly, both anti-IGF-II receptor antibodies and soluble IGF-II receptor have been reported to inhibit herpes simplex virus infection in vitro [15]. By inference, the recovery of several clones with mutations in genes involved in IGF-II expression/signalling pathway suggests that mutations in multiple genes may affect the same phenotype by acting on a common pathway.

Additionally, several of the inserted mutations encode proteins that are thought to participate in trafficking of cargo in cells, and may participate in various stages of virus infection. These include mutations in genes encoding three annexins (Anxa1, Anxa2, Anxa3) and calcyclin (*S100a6/S100a1*) – proteins that may bind to each other [16-18] – and mutations affecting cytoskeletal and cytoskeletal associated proteins (*Cald1*, Kif13b, *Mapt*, *Mkln1*, *Stmn1*, *Tm9sf4*, and *Tpm1*). Annexin-II associates with cytomegalovirus virions and anti-annexin-II antibodies have been found to prevent cytomegalovirus plaque formation [19]. Annexin-II is known to bind to several of the other gene products mutated in our library. These include annexin-I, calcyclin, and α-tropomysin (Supplementary Table [see Additional File 1]) [17,18,20].

One of the clones has a disruption of a novel cell receptor, OL-16, which is a member of the immunoglobulin superfamily [21,22]. A presumptive cellular receptor for reovirus, junctional adhesion molecule (*Jam*)-1 [22], has been shown to bind to all reovirus serotypes [23], whereas reovirus infection has been found to be host-cell specific [24]. OL-16 is expressed both in L-cells and in RIE-1 cells that can be infected by reovirus type 1 but not in murine erythroleukemia (MEL) cells that are resistant to infection by type 1 reovirus [25]. Forced expression of an OL-16 transgene in MEL cells increases their susceptibility to reovirus type 1 infection (J. Sheng and D. Rubin, unpublished results).

Several of the candidate genes have products that interact with either reovirus or with other viruses (Supplementary Table [see Additional File 1]). Cellular activities involved in post-transcriptional gene regulation may influence the processing or translation of virus transcripts. Two candidate genes participate in these processes. Eif3, part of a multi-subunit translation initiation complex, has been found to specifically bind the 5' end of hepatitis C and classical swine fever virus mRNA [26]. The *Cstf *64 KaD subunit, which affects polyadenylation of mRNA, can be cross-linked to the mRNA of herpes simplex mRNA in infected HeLa cell extracts [27]. Other candidate genes are associated with the interferon pathway and host inflammatory responses [28-30]. For example, IκBζ (MAIL), as a component of the NK-κB pathway, may directly or tangentially affect interferon production, inflammation, or apoptosis. In addition, one gene encodes 6-pyruvoyl-tetrahydropterin synthase (*Pts*), a major regulator of interferon activity [31] associated with inducible nitric oxide synthase (iNOS). iNOS levels within cells affects the efficiency of replication of many viruses, including the avian reoviruses [32,33].

Many of the targeted gene products have roles involving the Golgi or endosomal compartments (Figure 3), and additional genes play a role in differentiation or growth arrest. Of these, several are in the transforming growth factor (TGF)-β and NF-κB regulatory pathways, *Ppm1a *[34], *Madh7 *[35-37], *Ube1c *[38,39], and *Map3k7ip1 *[40-43] (Table 1, Supplementary Table [see Additional File 1]). In addition, subunits of the eif3 complex have been functionally linked to *Mapkbp1 *and the proteosome [44]. We have also disrupted a number of genes that participate in apoptosis (Supplementary Table [see Additional File 1]), and three disrupted genes affect *N*-linked protein glycosylation, a process that may affect compartmentalization of proteins or ligand interactions.

### Reovirus resistant cells have altered susceptibility to HSV-1

**Figure 4:**
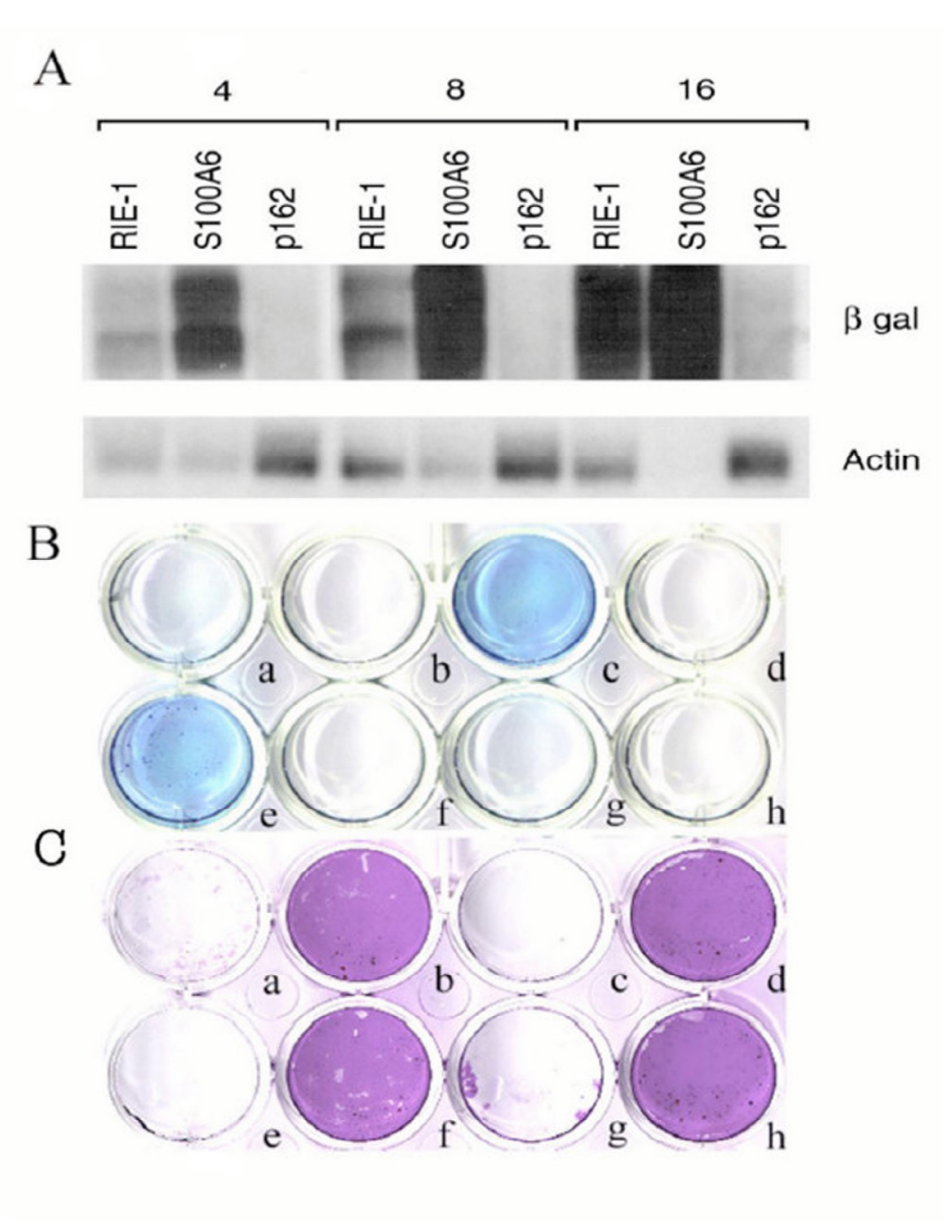
**HSV-1 infection is affected in cell clones selected as reovirus resistant **The level of transcription and translation of the reporter gene, lacZ, present in the immediate early genes of HSV-1 is shown in A and B. **A) **The level of expression of mRNA is shown at 4, 8 and 16 h following infection for a library of non-reovirus selected RIE-1 cells (L42), and two clones that disrupt the *Eif3s10 *(p162) and *S100a6 *genes. The cell clone with a disrupted *S100a6 *gene has a dramatic increase in HSV-1 expression with a concomitant decrease in cellular gene expression by 16 h. While there is more mRNA loaded in the lane with a disrupted *Eif3s10 *gene than is present in the other lanes, there is no evidence for HSV-1 expression in this cell until 16 h following infection. **B) **Translation of the *LacZ *reporter in the immediate early genes of HSV-1. At 8 hours following infection, the translation of the *LacZ *gene is dramatically increased in clones with mutant *S100a6 *and *Anxa2 *genes, barely detectable in the population of non-selected library cells (L42) and a cell clone that tags the *Aptx*^+^/*DnaJa1*^- ^genes, and is not evident in the other mutant clones. **C) **Cell survival was determined by gentian violet staining of cells at 72 hours. L42 cells, and clones with mutations in the *Aptx*^+^/*DnaJA1*, *annexin II*, and *S100a6 *genes lysed whereas clones with mutations in the *Eif3s10*, *Anax1*, *Mgat1*, and *Igfr2 *genes were resistant to lytic infection. a-library 42, non-selected; b-*Eif3s10*; c-calcyclin; d-*Anxa1*; e-*Anxa2*; f-*Mgat1*; g-*Aptx*^+^/*DnaJa1 *(negative strand); h-*Igf2r*

As several of the genes listed in the Supplementary Table [see Additional File 1] have been associated with herpes simplex-1 (HSV-1) replication, seven clones were tested for their susceptibility to HSV-1 infection [15,45]. These experiments utilized HSV-1(KOS)tk12, an infectious virus that expresses a *lac*Z reporter as an immediate-early gene [46]. Data representing seven clones with mutations that tag known genes are provided in Figure 4. Four clones, with mutations in the *Eif3s10*, *AnxaI*, *Mgat1*, and *Igf2r *genes, were resistant to HSV-I infection (Figure 4B,4C, b, d, f, and h) and there was a diminished capacity to express the immediate-early *lacZ *reporter gene. However, two of the clones (Figure 4B,4C, c and e) with mutations in genes encoding calcyclin and annexin-II, were more susceptible than the parental RIE-1 cells to HSV-1 infection and expressed higher levels of the immediate-early *lacZ *reporter gene. Representative clones that contain altered levels of HSV-1 immediate early gene expression are shown in Figure 4A. *LacZ *expression in cells containing a disrupted calcyclin (*S100a6*) gene was readily apparent 4 h following infection, whereas *lacZ *expression was barely detected in *Eif3s10 *mutant cells 16h following infection. In all cases, levels of *lacZ *expression correlated with susceptibility to HSV-1 infection, suggesting that resistance involved early steps in the viral lifecycle.

## Discussion

Candidate genes required for lytic reovirus infections were identified by tagged sequence mutagenesis, a process that permits rapid identification of genes disrupted by gene entrapment. Since virus-resistant mutants may arise by a variety of mechanisms, additional experiments are needed to demonstrate that individual genes disrupted by gene entrapment actually contribute to the reovirus-resistant phenotype. Even so, several lines of evidence suggest that the genes collectively identified by tagged sequence mutagenesis include cellular activities that participate in the virus lifecycle. First, reovirus-resistant clones were selected at higher frequencies from entrapment libraries than from non-mutagenized cells, suggesting that reovirus-resistant phenotypes were induced by gene trap mutagenesis. Second, the genes associated with reovirus resistance differed from genes targeted in an unselected manner in mouse ES cells. Known mutational hot spots of the U3NeoSV1 were under-represented, and a number of mutations associated with virus resistance appeared to cluster within specific cellular processes and/or affected different components of multi-protein complexes that are likely to play roles in the virus lifecycle. These include IGF-II expression/signalling (3 genes), cytoskeletal/vesicular/trafficking (20 genes), signalling pathways (11 genes), and apoptosis (4 genes). Finally, we recently demonstrated that the disruption of *Ctcf*, a transcriptional repressor of IGF-II, was directly responsible for reovirus-resistance. In particular, cells containing the *Ctcf *mutation express elevated levels IGF-II, while parental RIE1 cells forced to express IGF-II acquired high levels of reovirus resistance. The mutation in *Ctcf *was chosen for further analysis because it was one of 3 mutations affecting IGF-II expression and/or signalling [4]. By inference, the recovery of inserts affecting other genes in the IGF-II signalling pathway suggests that the same phenotype can be achieved through mutations in multiple genes in a common pathway. Taken together, these results suggest that the candidate genes identified by tagged sequence mutagenesis provide useful information to direct mechanistic studies of cellular processes that participate in the virus lifecycle.

These studies utilized a diploid cell line to select for reovirus resistance. Therefore, recessive phenotypes resulting from loss-of-function mutations are generally expected to require separate inactivation of genes carried on both autosomes. In principle this could occur through pre-existing heterozygosity or by loss of the unoccupied allele by one of several mechanisms such as gene conversion, non-disjunction or transcriptional repression. Several of the candidate genes discovered in these experiments are imprinted, and therefore may be anticipated to be mono-allelic in their expression, including the maternally imprinted *Igf2r*. Alternatively, mutations induced by gene entrapment may confer reovirus resistance as a result of gene dosage effects (e.g. haploinsufficiency). For example, recent data suggests that the most common genetic disease in Caucasians, cystic fibrosis, involves mutations in the ABC-cassette transporter protein, CFTR, that confer resistance to infection by *Salmonella typhi *[47]. Protection is afforded to persons heterozygous at this allele. Similarly, cyclosporin analogs, which affect P glycoprotein (a member of the ABC cassette transporters), inhibit the growth of *Cryptosporidium parum *[48]. Of note, one of the genes associated with reovirus resistance identified in the present study, *Pgy1 *(*Abcb1*), encodes P glycoprotein (Table 1, Supplementary Table [see Additional File 1]). Finally, while the U3NeoSV1 entrapment vector lacks the MuLV enhancer element, we cannot exclude the possibility that the phenotype observed was related to dominant mutations caused either by transcriptional activation of adjacent cellular genes or from the expression of truncated proteins with dominant-negative activity.

The circumstances that allow gene entrapment to disrupt the function of diploid genes illustrate that events secondary to provirus integration may be required for expression of some reovirus resistant phenotypes. Consequently, while the entrapment libraries were theoretically large enough (2 × 10^5 ^independent mutations) to disrupt all expressed genes, it seems unlikely that all the genes that are required for virus infection, and that can be targeted by tagged sequence mutagenesis, were identified in the present study.

Reovirus infection may induce apoptosis *in vivo *and *in vitro*, and the suppression of apoptosis enhances the survival of mice infected with reovirus type 3 [49,50]. Mutations associated with reovirus resistance included a number in proapototic genes including, *IκBζ *and *Bcl2l1*; however, the precise role of this pathway and the genes we have disrupted in modulating reovirus infectivity is unknown [51,52]. Therefore, while many genes are associated with known pathways, further studies will be required to understand the manner by which these pathways influence reovirus infection.

Genetic alterations giving rise to reovirus resistant clones have variable effects on HSV-1 replication, with some reovirus resistant clones showing enhanced HSV1 replication. The reasons for this are unknown, although each of these two viruses enter cells by different mechanisms. The early steps in HSV replication require entry into cells, release of the capsid with migration to the nucleus for which virus and cellular proteins play roles [53,54], whereas the entry of reovirus does not involve transit to the nucleus. Enhanced HSV-1 replication in clones containing mutations in the *S100a6 *(calcyclin) and *Anxa2 *genes was accompanied by a dramatic increase in immediate-early gene expression. This temporal enhancement of HSV-1 replication may reflect activities of calcyclin and annexin 2 proteins that suppress HSV-1 entry [55-57].

In addition to the clone with a mutation in *Anxa1*, clones with mutations in *Eif3s10*, *Mgat1*, and *Igf2r *also show decreases in transcription and translation of virus mRNA and cell death. Of these, mutations in the *Igf2r *are known to affect HSV replication [15,54,58]; whereas, association of HSV replication with proteins encoded by the *Eif3s10*, *Anxa1*, and *Mgat1 *are novel. These data suggest that some of the candidate genes discovered in clones surviving reovirus infection may affect common cellular processes that are used by other viruses.

Studies in a variety of systems indicate that resistance to infection may be found in nature or achieved in cultured cells. Results presented here support the hypothesis that pathways involved in reovirus infection can be identified through a functional genomics approach based upon insertional mutagenesis. Systematic selection of virus-resistant mutant cells in which the mutant gene can be easily identified may also identify targets for the development of anti-viral therapies. Drugs that disrupt cellular processes may circumvent the problem of virus resistance, generally observed with drugs against virus-encoded proteins. Moreover, since mutations associated with virus resistance are, by necessity, not lethal to the cell, drugs that target the same processes are not expected to have overtly toxic side effects. The fact that the resulting candidate genes may play roles in the replication of other viruses, suggests that different viruses may use similar host proteins for common steps required for virus entry, disassembly, transcription, translation, and reassembly. This view is supported by our studies with HSV-1 and by published reports implicating several of the genes disrupted in reovirus resistant cells (Supplementary Table [see Additional File 1]) in the replication of other viruses. Thus, mutant clones selected for resistance to lytic infection to one virus may provide targets for therapeutics that are active against other families of viruses.

The dramatic increase in the pace of the genome project has led to an explosion of information concerning the sequence of the genome of several species of animals and pathogenic organisms. However, most of the gene sequences have not been functionally ascribed with regard to host-parasite interactions. As there are approximately 30 to 50 × 10^3 ^mammalian genes, the definition of function will become the major task facing scientists interested in the relationship between host genes and viral disease over the next decade.

## Conclusions

Candidate host genes that participate in lytic virus infections were identified utilizing insertional mutagenesis. Mutant cell clones were recovered that lost their capacity to support virus replication, but were able to proliferate. There was enrichment for genes that were involved in particular metabolic or signalling pathways, with many of the genes being selected more than once from independently derived libraries of RIE-1 cells. Several of the gene products are known to bind to each other. These genes or their products, which are identified by this process of selection, may provide targets for therapeutic intervention.

## Methods

### RIE-1, L-Cells and Virus

Reovirus type 1, strain Lang, was initially obtained from Bernard N. Fields. Virus was passaged in L-cells and a third passaged stock was purified over a CsCl gradient as previously described and was used for these experiments [59]. To develop PI cell lines, RIE-1 cells were infected with reovirus type 1, at a multiplicity of infection (MOI) of 5, and surviving cells were maintained in Dulbecco's modification of Eagle's minimum essential medium (DMEM) (Irvine Scientific, Santa Ana, CA, USA). The herpes simplex virus (HSV)-1 clone, HSV-1 KOStk12, that expresses a reporter gene, lacZ, as an immediate-early gene [46] was a generous gift of Patricia Spear, Northwestern University, USA. For RIE-1 and L-cells, medium was supplemented with 10% fetal bovine serum, 2 mM per ml, L-Glutamine 100 units per ml, Penicillin, and 100 μg per ml Streptomycin (Irvine Scientific, Santa Ana, CA, USA) [complete medium]. In some experiments, serum was omitted from the medium. The continuance of cell monolayers following infection with reovirus or HSV-1 was determined by staining with gentian violet.

### Tagged sequence mutagenesis and selection for reovirus resistance

Following infection of RIE-1 cells with the U3neoSV1 vector, MOI of 0.1, mutagenized cells were selected for neomycin resistance in medium containing 1 mg/ml G418 sulfate (Clontech, Palo Alto, CA, USA) [6]. Twenty libraries of mutant RIE-1 cells, and one library of A549 human adenocarcinoma cells, each consisting of 10^4 ^gene entrapment events, were expanded until approximately 10^3 ^sibling cells represented each mutant clone. These cells were plated at a sub-confluent density and incubated in serum-free media for 3 days until they became quiescent, and infected with reovirus serotype 1, MOI of 35 plaque forming units (pfu) per cell. Eighteen hours following infection, the cells were detached with trypsin, and plated in DMEM medium containing 10% fetal bovine serum (FBS) (Hyclone Laboratories, Inc., Logan, Utah, USA). After 6 hrs, the medium was removed and cells were maintained in serum-free medium until only a few cells remained attached to the flask. On average, one to ten clones were recovered from a library consisting of 10^7 ^mutant cells, an enrichment for selected cells of six orders of magnitude. Cells that survived the selection were transferred to cell culture plates in media containing 10% FBS and cells were divided for extraction of DNA and cryopreservation.

### Transcription and translation of HSV-1 immediate early gene reporter

The transcription and translation of the HSV-1 immediate early gene reporter gene, *lacZ*, was determined by standard northern blot techniques and β-galactosidase assay, respectively.

### Generation of libraries of mutagenized RIE-1 cells

Libraries of mutagenized cells were infected with reovirus serotype-1, strain Lang, to select for clones resistant to lytic infection. Selection of virus-resistant clones was performed in serum-free medium to suppress the emergence of persistently infected (PI) cells [4]. This is important since PI cells, which arise by a process involving adaptive mutations in both the virus and the cell genomes [60], provide a means whereby RIE-1 cells can acquire virus resistance in the absence of cellular mutations. Uninfected RIE-1 cells undergo growth arrest, whereas PI RIE-1 cells are killed in serum-free medium.

### DNA sequence analysis

Genomic DNA immediately adjoining the 5' end of the proviral insert in each of 130 cell lines was cloned by plasmid rescue [6]. Approximately 300 to 600 base pairs of this flanking DNA were sequenced and compared with the non-redundant (nr) and expressed sequence tag (dbEST) nucleic acid databases [61]. The probability of a match with orthologous sequences in the databases varies due to interspecies variation, the amount of exon in the flanking DNA (in cases where the flanking DNA matches cDNA sequences), alternative splicing and sequencing errors. Matches with sequences in the database were considered potentially significant if probability score was <10^-5 ^and the sequence was non-repetitive. In most cases, the matching gene was in the same transcriptional orientation as the provirus. Moreover, matches involving cDNA sequences were co-linear across exons present in the flanking genomic DNA and diverged at splice sites. As indicated, virtually all of the genes identified had matches to murine, rat, or human gene sequences with p < 10^-10^.

## Authors' contributions

ELO, and JS conducted most of the laboratory work. HER provided the vectors and advice on their use. DHR discovered that persistently infected cells require serum to survive, allowing the selection of genetically resistant cell clones, and did the genetic analysis. Drs. Ruley and Rubin provided funding and supervision for the research, and prepared the manuscript. All authors have read and approved the final manuscript.

## Supplementary Material

Additional File 1 **Genes associated with resistance to lytic reovirus infection identified by tagged sequence mutagenesis **A list of previously named genes disrupted by the insertional mutagen, U3neoSV1, and recovered in cell clones resistant to lytic infection is provided. The rat mRNA and vector insertion site accession number, rat chromosome location, human homologue chromosome location, link to the NCBI Entrez Gene and NCBI Nucleotide databases, and known virus interactions are listed. Click here for file
