# Supplementary material for: Discovery of mammalian genes that participate in virus infection
Source: BMC Cell Biol. 2004 Nov 2;5:41. doi: 10.1186/1471-2121-5-41 (PMC534806; doi:10.1186/1471-2121-5-41)
Supplement: Additional File 1 — Genes associated with resistance to lytic reovirus infection identified by tagged sequence mutagenesis A list of previously named genes disrupted by the insertional mutagen, U3neoSV1, and recovered in cell clones resistant to lytic infection is provided. The rat mRNA and vector insertion site accession number, rat chromosome location, human homologue chromosome location, link to the NCBI Entrez Gene and NCBI Nucleotide databases, and known virus interactions are listed. [file 1471-2121-5-41-S1.doc]

Additional File 1. Genes associated with resistance to lytic reovirus infection identified by tagged sequence mutagenesis

Mutagenized libraries of RIE-1 cells were infected with reovirus type 1 and selected for resistance to lytic infection. The genes that comprise this list are previously named genes with the exception of several genes that have not been named, such as *Chr10orf3*, as it was recovered independently from 3 separately prepared libraries screened for resistance to reovirus. In other instances, the vector may disrupt two genes, one of which is located off the negative strand in relation to the vector (-). In the first column, **Genes**, the yellow background indicates recovery of two or more clones independently obtained with the vector inserted into the same gene. In the column, **Definitions**, a short description of the gene is provided. The assignment of chromosomal location on the rat (**R Chr**) and human (**H Chr**) genomes, along with the **Rat Genbank Accession # mRNA** and the **Genbank Accession # Insert** are provided. LocusLink or Entrez Gene numbers (**Entrez Gene for Rat Gene**) provide links to the National Library of Medicine [1] as well as direct hyperlinks to the National Library of Medicine from the web-based publication. Similarly, the accession numbers for the genomic insertion sites of the vector are provided with the **Rat Genbank Accession # mRNA** [2]. In the column, **Virus interaction** includes viruses known to physically interact with or require the gene products of a candidate gene (ADV: adenovirus, CSFV: classical swine fever virus, Friend leukemia virus, FLV, IFAV: influenza A virus, hCMV: human cytomegalovirus, HCV: hepatitis C virus, human herpes virus-8: HHV8, HEV: hepatitis E virus, HSV: herpes simplex virus, HcoV: Human coronavirus, reo: polio: poliovirus, reovirus, SV40: simian virus 40, VSV: vesicular stomatitis virus, VV, vaccinia virus).

| **Genes** | **Definition** | **R Chr** | **H Chr** | **Rat Genbank Accession # mRNA** | **Genbank Accession # Insert** | **Entrez Gene for Rat Gene** | **Virus Interactions** |
| --- | --- | --- | --- | --- | --- | --- | --- |
| *Loc310836 (Abca4)* | ATP-binding cassette, subfamily A (ABC1), member 4 | 2q41 | 1p22.1-p21 | XP_241525 | [AR228076](http://www.ncbi.nlm.nih.gov/entrez/viewer.fcgi?db=nucleotide&val=27266822)  [BD069426](http://www.ncbi.nlm.nih.gov/entrez/viewer.fcgi?db=nucleotide&val=22615029) | [310836](http://www.ncbi.nlm.nih.gov/entrez/query.fcgi?db=gene&cmd=Retrieve&dopt=Graphics&list_uids=310836) |  |
| *Anxa1* | annexin A1 | 1q51 | 9q12-q21.2 | U25159 | [AR228072](http://www.ncbi.nlm.nih.gov/entrez/viewer.fcgi?db=nucleotide&val=27266818)  [BD069422](http://www.ncbi.nlm.nih.gov/entrez/viewer.fcgi?db=nucleotide&val=22615025) | [25380](http://www.ncbi.nlm.nih.gov/entrez/query.fcgi?db=gene&cmd=Retrieve&dopt=Graphics&list_uids=25380) | HSV [3] |
| *Anxa2* | annexin A2 | 8q24 | 15q21-q22 | NM_019905 | [AR228047](http://www.ncbi.nlm.nih.gov/entrez/viewer.fcgi?db=nucleotide&val=27266793)  [BD069397](http://www.ncbi.nlm.nih.gov/entrez/viewer.fcgi?db=nucleotide&val=22615000) [BD078939](http://www.ncbi.nlm.nih.gov/entrez/viewer.fcgi?db=nucleotide&val=22624542) | [56611](http://www.ncbi.nlm.nih.gov/entrez/query.fcgi?db=gene&cmd=Retrieve&dopt=Graphics&list_uids=56611) | CMV  [4, 5] |
| *Anx3* | annexin A3 | 14p22 | 4q13-q22 | NM_012823 | [BD079025](http://www.ncbi.nlm.nih.gov/entrez/viewer.fcgi?db=nucleotide&val=22624628) | [25291](http://www.ncbi.nlm.nih.gov/entrez/query.fcgi?db=gene&cmd=Retrieve&dopt=Graphics&list_uids=25291) |  |
| *Aptx^-^/ dnaja1 [Hsj2]* | aprataxin/DnaJ (Hsp40) homolog, subfamily A, member 1 | 5q22 | 9p13.3 | NW_07454 | [BD078944](http://www.ncbi.nlm.nih.gov/entrez/viewer.fcgi?db=nucleotide&val=22624547)  [BD078943](http://www.ncbi.nlm.nih.gov/entrez/viewer.fcgi?db=nucleotide&val=22624546)  [BD078945](http://www.ncbi.nlm.nih.gov/entrez/viewer.fcgi?db=nucleotide&val=22624548) | [259271](http://www.ncbi.nlm.nih.gov/entrez/query.fcgi?db=gene&cmd=Retrieve&dopt=Graphics&list_uids=259271)  [65028](http://www.ncbi.nlm.nih.gov/entrez/query.fcgi?db=gene&cmd=Retrieve&dopt=Graphics&list_uids=65028) |  |
| *Atp6v0c* | ATPase, H+ transporting, lysosomal 16kDa, V0 subunit c | 10q12 | 16p13.3 | NC_000016 | [AR228008](http://www.ncbi.nlm.nih.gov/entrez/viewer.fcgi?db=nucleotide&val=27266754)  [BD069358](http://www.ncbi.nlm.nih.gov/entrez/viewer.fcgi?db=nucleotide&val=22614961) | [170667](http://www.ncbi.nlm.nih.gov/entrez/query.fcgi?db=gene&cmd=Retrieve&dopt=Graphics&list_uids=170667) | reo, IFAV, VSV, HIV.  SFV  [6-10] |
| *Bcl2l1* | Bcl2-like1 | 3q41.2 | 20q11.21 | NP_238186 | [BD078973](http://www.ncbi.nlm.nih.gov/entrez/viewer.fcgi?db=nucleotide&val=22624576)  [BD079032](http://www.ncbi.nlm.nih.gov/entrez/viewer.fcgi?db=nucleotide&val=22624635) | [24888](http://www.ncbi.nlm.nih.gov/entrez/query.fcgi?db=gene&cmd=Retrieve&dopt=Graphics&list_uids=24888) |  |
| *Brd2* | bromodomain-containing 2 | 20p12 | 6p21.3 | XP_238186 | [BD078987](http://www.ncbi.nlm.nih.gov/entrez/viewer.fcgi?db=nucleotide&val=22624590) | [294276](http://www.ncbi.nih.gov/LocusLink/LocRpt.cgi?l=294276) | HHV8 [11] |
| *Brd3/*  *Wdr5* | Bromodomain-containing 3/  WD repeat domain 5 | 3p12 | 9q34 | XP_342398  XP_342397 | [AR228036](http://www.ncbi.nlm.nih.gov/entrez/viewer.fcgi?db=nucleotide&val=27266782)  [BD069386](http://www.ncbi.nlm.nih.gov/entrez/viewer.fcgi?db=nucleotide&val=22614989) | [362093](http://www.ncbi.nlm.nih.gov/entrez/query.fcgi?db=gene&cmd=retrieve&dopt=default&list_uids=362093) |  |
| *C9orf119^+^/ Golga2-* | gene of unknown function and Golgi autoantigen, Golgin subfamily a, 2 | 3p11 | 9q34.13 | NW_047652  NM_022596 | [BD078967](http://www.ncbi.nlm.nih.gov/entrez/viewer.fcgi?db=nucleotide&val=22624570) | [375757](http://www.ncbi.nlm.nih.gov/entrez/query.fcgi?db=gene&cmd=Retrieve&dopt=Graphics&list_uids=375757)  [64528](http://www.ncbi.nlm.nih.gov/entrez/query.fcgi?db=gene&cmd=retrieve&dopt=default&list_uids=64528) |  |
| *C10orf3* | chromosome 10 open reading frame 3 | 1q54 | 10q23.33 | XP_220034 | [AR228054](http://www.ncbi.nlm.nih.gov/entrez/viewer.fcgi?db=nucleotide&val=27266800)  [BD069404](http://www.ncbi.nlm.nih.gov/entrez/viewer.fcgi?db=nucleotide&val=22615007) [BD078978](http://www.ncbi.nlm.nih.gov/entrez/viewer.fcgi?db=nucleotide&val=22624581)  [AR228049](http://www.ncbi.nlm.nih.gov/entrez/viewer.fcgi?db=nucleotide&val=27266795)  [BD069399](http://www.ncbi.nlm.nih.gov/entrez/viewer.fcgi?db=nucleotide&val=22615002) | [55165](http://www.ncbi.nlm.nih.gov/LocusLink/LocRpt.cgi?l=55165) |  |
| *Cald1* | caldesmon 1 | 4q22 | 7q33 | NM_013146 | [BD078958](http://www.ncbi.nlm.nih.gov/entrez/viewer.fcgi?db=nucleotide&val=22624561) | [25687](http://www.ncbi.nlm.nih.gov/entrez/query.fcgi?db=gene&cmd=Retrieve&dopt=Graphics&list_uids=25687) |  |
| *Calm2* | calmodulin 2 (phosphorylase kinase, delta) | 6q11-q12 | 2p21 | NM_017326 | [BD078921](http://www.ncbi.nlm.nih.gov/entrez/viewer.fcgi?db=nucleotide&val=22624524) | [50663](http://www.ncbi.nlm.nih.gov/entrez/query.fcgi?db=gene&cmd=Retrieve&dopt=Graphics&list_uids=50663) |  |
| *Celsr2* | cadherin, EGF LAG seven-pass G-type receptor 2 | 4q11 | 1p21 | NW_047687 | [AR228032](http://www.ncbi.nlm.nih.gov/entrez/viewer.fcgi?db=nucleotide&val=27266778)  [AR228027](http://www.ncbi.nlm.nih.gov/entrez/viewer.fcgi?db=nucleotide&val=27266773)  [BD069377](http://www.ncbi.nlm.nih.gov/entrez/viewer.fcgi?db=nucleotide&val=22614980)  [BD069382](http://www.ncbi.nlm.nih.gov/entrez/viewer.fcgi?db=nucleotide&val=22614985) | [83465](http://www.ncbi.nih.gov/LocusLink/LocRpt.cgi?l=83465) |  |
| *Clr-f* | killer cell lectin-like receptor F1 | 4q12 | N/A | XM_232399 | [BD078982](http://www.ncbi.nlm.nih.gov/entrez/viewer.fcgi?db=nucleotide&val=22624585)  [AR228038](http://www.ncbi.nlm.nih.gov/entrez/viewer.fcgi?db=nucleotide&val=27266784) | [312745](http://www.ncbi.nlm.nih.gov/entrez/query.fcgi?db=gene&cmd=retrieve&dopt=default&list_uids=312745) |  |
| *Copg2/*  *Tsga13* | coatomer protein complex, subunit gamma 2/ testis specific gene A13- variegated monoallelic expression 1 | 4q22 | 7q32 | NW_047355  XP_231582 | [AR228051](http://www.ncbi.nlm.nih.gov/entrez/viewer.fcgi?db=nucleotide&val=27266797) [BD078955](http://www.ncbi.nlm.nih.gov/entrez/viewer.fcgi?db=nucleotide&val=22624558)  [BD078941](http://www.ncbi.nlm.nih.gov/entrez/viewer.fcgi?db=nucleotide&val=22624544)  [BD069401](http://www.ncbi.nlm.nih.gov/entrez/viewer.fcgi?db=nucleotide&val=22615004)  [BD078988](http://www.ncbi.nlm.nih.gov/entrez/viewer.fcgi?db=nucleotide&val=22624591) | [301742](http://www.ncbi.nlm.nih.gov/entrez/query.fcgi?db=gene&cmd=Retrieve&dopt=Graphics&list_uids=301742)  [312203](http://www.ncbi.nlm.nih.gov/entrez/query.fcgi?db=gene&cmd=Retrieve&dopt=Graphics&list_uids=312203) |  |
| *Csmd2* | CUB and sushi multiple domains 2 | 5q36 | 1p35.1-p34.3 | XP_232753 | [BD079033](http://www.ncbi.nlm.nih.gov/entrez/viewer.fcgi?db=nucleotide&val=22624636) | [313040](http://www.ncbi.nlm.nih.gov/entrez/query.fcgi?db=gene&cmd=Retrieve&dopt=Graphics&list_uids=313040) |  |
| *Cstf2t* | cleavage stimulation factor, 3' pre-RNA, subunit 2, 64kDa | 1q52 | xq22.1 | NW_047565 | [BD078960](http://www.ncbi.nlm.nih.gov/entrez/viewer.fcgi?db=nucleotide&val=22624563)  [BD078958](http://www.ncbi.nlm.nih.gov/entrez/viewer.fcgi?db=nucleotide&val=22624561) | [309338](http://www.ncbi.nlm.nih.gov/entrez/query.fcgi?db=gene&cmd=retrieve&dopt=default&list_uids=309338) | HSV, SV40, ADV, papilloma [12-15] |
| *Ctcf* | CCCTC-binding factor (zinc finger protein) | 19q12 | 16q21-q22.3 | NP_114012 | [AR228009](http://www.ncbi.nlm.nih.gov/entrez/viewer.fcgi?db=nucleotide&val=27266755)  [BD069366](http://www.ncbi.nlm.nih.gov/entrez/viewer.fcgi?db=nucleotide&val=22614969) | [83726](http://www.ncbi.nlm.nih.gov/entrez/query.fcgi?db=gene&cmd=Retrieve&dopt=Graphics&list_uids=83726) |  |
| *Cutl1/*  *Mylc2pl*^+^ | cut-like 1, CCAAT displacement protein (Drosophila) | 12q12 | 7q22.1 | XP_341054  XP_344098 | [BD079017](http://www.ncbi.nlm.nih.gov/entrez/viewer.fcgi?db=nucleotide&val=22624620)  [BD078994](http://www.ncbi.nlm.nih.gov/entrez/viewer.fcgi?db=nucleotide&val=22624597) | [116639](http://www.ncbi.nlm.nih.gov/entrez/query.fcgi?db=gene&cmd=Retrieve&dopt=Graphics&list_uids=116639)  [363900](http://www.ncbi.nlm.nih.gov/entrez/query.fcgi?db=gene&cmd=Retrieve&dopt=Graphics&list_uids=363900) |  |
| *Dlx2* | distal-less homeo box 2 similar to TES-1 homeobox | 3q21 | 2q32 | XP_230986 | [BD079037](http://www.ncbi.nlm.nih.gov/entrez/viewer.fcgi?db=nucleotide&val=22624640) | [296499](http://www.ncbi.nlm.nih.gov/entrez/query.fcgi?db=gene&cmd=Retrieve&dopt=Graphics&list_uids=296499) |  |
| *Dnaja1* | DnaJ (Hsp40) homolog, subfamily A, member 1 | 5q22 | 9p13-p12 | NP_075223 | [BD078944](http://www.ncbi.nlm.nih.gov/entrez/viewer.fcgi?db=nucleotide&val=22624547) | [65028](http://www.ncbi.nlm.nih.gov/entrez/query.fcgi?db=gene&cmd=Retrieve&dopt=Graphics&list_uids=65028) |  |
| *Dre1* | Dre1 protein | 11q23 | 3q27.1 | NM_181473 | [AR228014](http://www.ncbi.nlm.nih.gov/entrez/viewer.fcgi?db=nucleotide&val=27266760)  [BD069364](http://www.ncbi.nlm.nih.gov/entrez/viewer.fcgi?db=nucleotide&val=22614967)  [BD078933](http://www.ncbi.nlm.nih.gov/entrez/viewer.fcgi?db=nucleotide&val=22624536) | [303803](http://www.ncbi.nlm.nih.gov/entrez/query.fcgi?db=gene&cmd=Retrieve&dopt=Graphics&list_uids=303803) |  |
| *E2ig2* | Estrogen induced gene 2 | 1q32 | 11q13.3 | NP_057649 | [BD078972](http://www.ncbi.nlm.nih.gov/entrez/viewer.fcgi?db=nucleotide&val=22624575) | [51287](http://www.ncbi.nlm.nih.gov/LocusLink/LocRpt.cgi?l=51287) |  |
| *Eif3s10* | Elongation initiation factor 3 subunit 10 | 1q55 | 10q26 | NW_047570 | [AR228011](http://www.ncbi.nlm.nih.gov/entrez/viewer.fcgi?db=nucleotide&val=27266757)  [BD069361](http://www.ncbi.nlm.nih.gov/entrez/viewer.fcgi?db=nucleotide&val=22614964) | [292148](http://www.ncbi.nlm.nih.gov/LocusLink/LocRpt.cgi?l=292148) | HCV,  polio  CSFV  [16-18] |
| *Erbb2ip* | Erbb2 interacting protein | 2q12 | 5q13.1 | XP_345149 | [AR228015](http://www.ncbi.nlm.nih.gov/entrez/viewer.fcgi?db=nucleotide&val=27266761)  [BD069365](http://www.ncbi.nlm.nih.gov/entrez/viewer.fcgi?db=nucleotide&val=22614968) | [55914](http://www.ncbi.nlm.nih.gov/LocusLink/LocRpt.cgi?l=55914) |  |
| *Fer1l3* | fer-1-like protein 3, myoferlin | 1q53 | 10q24 | NW_047565 | [AR228049](http://www.ncbi.nlm.nih.gov/entrez/viewer.fcgi?db=nucleotide&val=27266795)  [BD069399](http://www.ncbi.nlm.nih.gov/entrez/viewer.fcgi?db=nucleotide&val=22615002) | [26509](http://www.ncbi.nlm.nih.gov/LocusLink/LocRpt.cgi?l=26509) |  |
| *Fkbp8* | FK506 binding protein 8, 38kDa | 16p14 | 19p12 | XP_214316 | | [BD079018](http://www.ncbi.nlm.nih.gov/entrez/viewer.fcgi?db=nucleotide&val=22624621)  [BD078996](http://www.ncbi.nlm.nih.gov/entrez/viewer.fcgi?db=nucleotide&val=22624599)  [BD079016](http://www.ncbi.nlm.nih.gov/entrez/viewer.fcgi?db=nucleotide&val=22624619)  [BD078993](http://www.ncbi.nlm.nih.gov/entrez/viewer.fcgi?db=nucleotide&val=22624596) | | --- | | [23770](http://www.ncbi.nlm.nih.gov/LocusLink/LocRpt.cgi?l=23770) | HIV [19] |
| *Fusip1* | FUS interacting protein (serine-arginine rich) 1 | 5q36 | 1p36.11 | XP_342949 | [BD079009](http://www.ncbi.nlm.nih.gov/entrez/viewer.fcgi?db=nucleotide&val=22624612) | [362630](http://www.ncbi.nlm.nih.gov/entrez/query.fcgi?db=gene&cmd=Retrieve&dopt=Graphics&list_uids=362630) | ADV [20] |
| *Gas5* | Growth arrest specific 5 | 13q21 | 1q23.3 | U77829 | [AR228010](http://www.ncbi.nlm.nih.gov/entrez/viewer.fcgi?db=nucleotide&val=27266756)  [BD069360](http://www.ncbi.nlm.nih.gov/entrez/viewer.fcgi?db=nucleotide&val=22614963) | [14455](http://www.ncbi.nlm.nih.gov/LocusLink/LocRpt.cgi?l=14455) |  |
| *Gata4* | GATA binding protein 4 | 15p12 | 8p23.1-p22 | NW_047454 | [AR228023](http://www.ncbi.nlm.nih.gov/entrez/viewer.fcgi?db=nucleotide&val=27266769)  [BD069373](http://www.ncbi.nlm.nih.gov/entrez/viewer.fcgi?db=nucleotide&val=22614976)  [AR228024](http://www.ncbi.nlm.nih.gov/entrez/viewer.fcgi?db=nucleotide&val=27266770) | [2626](http://www.ncbi.nlm.nih.gov/LocusLink/LocRpt.cgi?l=2626) |  |
| *Grb2* | Growth factor receptor bound protein 2 | 10q32.2 | 17q24-q25 | NP_110473 | In process | [81504](http://www.ncbi.nlm.nih.gov/entrez/query.fcgi?db=gene&cmd=retrieve&dopt=default&list_uids=81504) | HCV, HEV, FLV, VV,  [21-24] |
| *Gtf2e1/*  *Rabl3* | General transcription factor IIE, polypeptide 1, alpha 56kDa;  RAB, member of RAS oncogene family-like 3 | 11q22 | 3q21-q24 | XP_221426  XP_340993 | [BD078975](http://www.ncbi.nlm.nih.gov/entrez/viewer.fcgi?db=nucleotide&val=22624578) | [2960](http://www.ncbi.nih.gov/LocusLink/LocRpt.cgi?l=2960)  [360720](http://www.ncbi.nlm.nih.gov/entrez/query.fcgi?db=gene&cmd=Retrieve&dopt=Graphics&list_uids=360720) | HIV [25, 26] |
| *HM13* | histocompatibility (minor) 13, human gene from A549 cell library | 3q41 | 20q11.21 | NM_030789 | In process | [81502](http://www.ncbi.nlm.nih.gov/entrez/query.fcgi?db=gene&cmd=retrieve&dopt=default&list_uids=81502) |  |
| *HNRPL* | heterogeneous nuclear ribonucleoprotein L- human gene from A549 cell library | 1q21 | 19q13.2 | NP_001524 | In process | [3191](http://www.ncbi.nlm.nih.gov/LocusLink/LocRpt.cgi?l=3191) |  |
| *Hoxc13* | Homeo box C13 | 7q36 | 12q13.3 | XP_345881 | [BD078923](http://www.ncbi.nlm.nih.gov/entrez/viewer.fcgi?db=nucleotide&val=22624526) | [366995](http://www.ncbi.nlm.nih.gov/entrez/query.fcgi?db=gene&cmd=retrieve&dopt=default&list_uids=366995) |  |
| *Hp1-bp74* | heterochromatin protein 1, binding protein 74 | 5q36 | 1p36.13 | NM_199108 | [AR228022](http://www.ncbi.nlm.nih.gov/entrez/viewer.fcgi?db=nucleotide&val=27266768)  [BD069372](http://www.ncbi.nlm.nih.gov/entrez/viewer.fcgi?db=nucleotide&val=22614975) | [313647](http://www.ncbi.nlm.nih.gov/entrez/query.fcgi?db=gene&cmd=retrieve&dopt=default&list_uids=313647) |  |
| *Hspc135^+^/*  *Mox2r* | HSPC135 protein homologue/  Cd200 receptor 2 | 11q21 | 3q13.2 | [XP_340986](http://www.ncbi.nlm.nih.gov/entrez/query.fcgi?dopt=GenPept&cmd=Retrieve&db=protein&list_uids=34869140)  NP_076443 | [AR228059](http://www.ncbi.nlm.nih.gov/entrez/viewer.fcgi?db=nucleotide&val=27266805)  [BD069409](http://www.ncbi.nlm.nih.gov/entrez/viewer.fcgi?db=nucleotide&val=22615012) | [360714](http://www.ncbi.nlm.nih.gov/entrez/query.fcgi?db=gene&cmd=retrieve&dopt=default&list_uids=360714)  [131450](http://www.ncbi.nlm.nih.gov/LocusLink/LocRpt.cgi?l=131450) |  |
| *Id3* | Inhibitor of DNA binding 3, dominant negative helix-loop-helix protein | 5q36 | 1p36.13-p36.12 | NP_037190 | [BD078974](http://www.ncbi.nlm.nih.gov/entrez/viewer.fcgi?db=nucleotide&val=22624577)  [BD078924](http://www.ncbi.nlm.nih.gov/entrez/viewer.fcgi?db=nucleotide&val=22624527)  [BD079042](http://www.ncbi.nlm.nih.gov/entrez/viewer.fcgi?db=nucleotide&val=22624645) | [25585](http://www.ncbi.nih.gov/LocusLink/LocRpt.cgi?l=25585) |  |
| *Igf2r* | Insulin-like growth factor 2 receptor | 1q11 | 6q26 | NW_047553 | [BD078980](http://www.ncbi.nlm.nih.gov/entrez/viewer.fcgi?db=nucleotide&val=22624583) | [25151](http://www.ncbi.nlm.nih.gov/entrez/query.fcgi?db=gene&cmd=Retrieve&dopt=Graphics&list_uids=25151) | HSV  [27-29] |
| IκBζ (MAIL) | Molecule possessing ankyrin repeats induced by lipopolysaccharide | 11q12 | 3p12-q12 | NW_047355 | [BD079015](http://www.ncbi.nlm.nih.gov/entrez/viewer.fcgi?db=nucleotide&val=22624618)  [BD078992](http://www.ncbi.nlm.nih.gov/entrez/viewer.fcgi?db=nucleotide&val=22624595) | [64332](http://www.ncbi.nlm.nih.gov/LocusLink/LocRpt.cgi?l=64332) |  |
| *Jak1* | Tyrosine-protein kinase JAK1 | 5q33 | 1p32.3-p31.3 | XP_342873 | [BD078969](http://www.ncbi.nlm.nih.gov/entrez/viewer.fcgi?db=nucleotide&val=22624572)  [BD079044](http://www.ncbi.nlm.nih.gov/entrez/viewer.fcgi?db=nucleotide&val=22624647) | [362552](http://www.ncbi.nlm.nih.gov/entrez/query.fcgi?db=gene&cmd=Retrieve&dopt=Graphics&list_uids=362552) | HBV [30] |
| *Ki-67* | proliferation related antigen | 2q26 | 10q25 | XP_227096 | [AR228033](http://www.ncbi.nlm.nih.gov/entrez/viewer.fcgi?db=nucleotide&val=27266779)  [BD069383](http://www.ncbi.nlm.nih.gov/entrez/viewer.fcgi?db=nucleotide&val=22614986) | [310382](http://www.ncbi.nlm.nih.gov/entrez/query.fcgi?db=gene&cmd=Retrieve&dopt=Graphics&list_uids=310382) |  |
| *Kif13b* | guanylate kinase associated kinesin | 15p12 | 8p12 | XP_224288 | [BD078976](http://www.ncbi.nlm.nih.gov/entrez/viewer.fcgi?db=nucleotide&val=22624579) | [305967](http://www.ncbi.nlm.nih.gov/LocusLink/LocRpt.cgi?l=305967) |  |
| *Klhl6* | kelch-like 6 | 11q23 | 3q27.3 | NW_047358 | [AR228020](http://www.ncbi.nlm.nih.gov/entrez/viewer.fcgi?db=nucleotide&val=27266766)  [BD069370](http://www.ncbi.nlm.nih.gov/entrez/viewer.fcgi?db=nucleotide&val=22614973)  [BD078933](http://www.ncbi.nlm.nih.gov/entrez/viewer.fcgi?db=nucleotide&val=22624536)  [AR228014](http://www.ncbi.nlm.nih.gov/entrez/viewer.fcgi?db=nucleotide&val=27266760) | [89857](http://www.ncbi.nlm.nih.gov/LocusLink/LocRpt.cgi?l=89857) |  |
| *Lipc/*  LOC363090 | lipase, member H  glyceraldehyde-3-phosphate dehydrogenase (phosphorylating) | 8q24 | 15q21-q23 | NM_012597XP_343422 | [AR228018](http://www.ncbi.nlm.nih.gov/entrez/viewer.fcgi?db=nucleotide&val=27266764)  [BD069368](http://www.ncbi.nlm.nih.gov/entrez/viewer.fcgi?db=nucleotide&val=22614971) [AR228046](http://www.ncbi.nlm.nih.gov/entrez/viewer.fcgi?db=nucleotide&val=27266792)  [BD069396](http://www.ncbi.nlm.nih.gov/entrez/viewer.fcgi?db=nucleotide&val=22614999) | [24538](http://www.ncbi.nlm.nih.gov/entrez/query.fcgi?db=gene&cmd=Retrieve&dopt=Graphics&list_uids=24538)  [363090](http://www.ncbi.nlm.nih.gov/entrez/query.fcgi?db=gene&cmd=Retrieve&dopt=Graphics&list_uids=363090) |  |
| *Madh7* | MAD, mothers against decapentaplegic homolog 7 (Drosophila) | 18q12.3 | 18q21.1 | NW_047516 | [BD078950](http://www.ncbi.nlm.nih.gov/entrez/viewer.fcgi?db=nucleotide&val=22624553) | [4092](http://www.ncbi.nlm.nih.gov/LocusLink/LocRpt.cgi?l=4092) |  |
| *Map3k7ip1* | Mitogen-activated protein kinase kinase kinase 7 interacting protein 1 | 7q34 | 22q13.1 | NW_047780 | [BD078985](http://www.ncbi.nlm.nih.gov/entrez/viewer.fcgi?db=nucleotide&val=22624588) | [10454](http://www.ncbi.nlm.nih.gov/LocusLink/LocRpt.cgi?l=10454) |  |
| *Mapt* | Microtubule-associated protein TAU | 10q32.1 | 17q21.1 | NM_017212 | [AR228033](http://www.ncbi.nlm.nih.gov/entrez/viewer.fcgi?db=nucleotide&val=27266779)  [BD069383](http://www.ncbi.nlm.nih.gov/entrez/viewer.fcgi?db=nucleotide&val=22614986) | [360248](http://www.ncbi.nlm.nih.gov/entrez/query.fcgi?db=gene&cmd=Retrieve&dopt=Graphics&list_uids=360248) |  |
| *Mgat1* | mannosyl (alpha-1,3-)-glycoprotein beta-1,2-N-acetyl-glucosaminyl-transferase | 10q21 | 5q35 | NW_047334 | [AR228012](http://www.ncbi.nlm.nih.gov/entrez/viewer.fcgi?db=nucleotide&val=27266758)  [BD069362](http://www.ncbi.nlm.nih.gov/entrez/viewer.fcgi?db=nucleotide&val=22614965)  [BD079013](http://www.ncbi.nlm.nih.gov/entrez/viewer.fcgi?db=nucleotide&val=22624616)  [BD078952](http://www.ncbi.nlm.nih.gov/entrez/viewer.fcgi?db=nucleotide&val=22624555) | [81519](http://www.ncbi.nlm.nih.gov/entrez/query.fcgi?db=gene&cmd=Retrieve&dopt=Graphics&list_uids=81519) | VSV[6] |
| *Mical2* | flavoprotein oxidoreductase | 1q33 | 11p15.3 | NP_872610 | [AR228031](http://www.ncbi.nlm.nih.gov/entrez/viewer.fcgi?db=nucleotide&val=27266777)  [BD069381](http://www.ncbi.nlm.nih.gov/entrez/viewer.fcgi?db=nucleotide&val=22614984)  [BD078931](http://www.ncbi.nlm.nih.gov/entrez/viewer.fcgi?db=nucleotide&val=22624534) | [365352](http://www.ncbi.nlm.nih.gov/entrez/query.fcgi?db=gene&cmd=Retrieve&dopt=Graphics&list_uids=365352) |  |
| *Numb* | putative inhibitor of Notch signalling | 6q31 | 14q24.3 | XP_234394 | [AR228078](http://www.ncbi.nlm.nih.gov/entrez/viewer.fcgi?db=nucleotide&val=27266824) | [29419](http://www.ncbi.nlm.nih.gov/entrez/query.fcgi?db=gene&cmd=Retrieve&dopt=Graphics&list_uids=29419) |  |
| *Ocil* | osteoclast inhibitory lectin C-type | 4q32 | 12p13 | XP_342770 | [AR228081](http://www.ncbi.nlm.nih.gov/entrez/viewer.fcgi?db=nucleotide&val=27266827)  [BD079024](http://www.ncbi.nlm.nih.gov/entrez/viewer.fcgi?db=nucleotide&val=22624627)  [BD069431](http://www.ncbi.nlm.nih.gov/entrez/viewer.fcgi?db=nucleotide&val=22615034)  [BD078961](http://www.ncbi.nlm.nih.gov/entrez/viewer.fcgi?db=nucleotide&val=22624564)  [BD078951](http://www.ncbi.nlm.nih.gov/entrez/viewer.fcgi?db=nucleotide&val=22624554) | [113937](http://www.ncbi.nih.gov/LocusLink/LocRpt.cgi?l=113937) |  |
| *OL16 [Asam]* | adipocyte specific protein 5 and splice variant ol-16 | 8q22 | 11q24.1 | NM_173154 | In process | [286939](http://www.ncbi.nih.gov/LocusLink/LocRpt.cgi?l=286939) |  |
| *Pde4b^-^/* | phosphodiesterase 4B, cAMP-specific (phosphodiesterase E4 dunce homolog, Drosophila) | 5q32 | 1p31 | NP_058727 | [BD078990](http://www.ncbi.nlm.nih.gov/entrez/viewer.fcgi?db=nucleotide&val=22624593) | [24626](http://www.ncbi.nlm.nih.gov/entrez/query.fcgi?db=gene&cmd=Retrieve&dopt=Graphics&list_uids=24626) |  |
| *Pgy1- (Abcb1)/* | ATP-binding cassette, sub-family B (MDR/TAP), member 1 | 4q12 | 16q12.1 | NM_012623 | [BD078989](http://www.ncbi.nlm.nih.gov/entrez/viewer.fcgi?db=nucleotide&val=22624592) | [24646](http://www.ncbi.nlm.nih.gov/entrez/query.fcgi?db=gene&cmd=Retrieve&dopt=Graphics&list_uids=24646) |  |
| *Prss11* | protease, serine, 11 (IGF binding) | 1q37 | 10q26.3 | NP_113909 | [BD069425](http://www.ncbi.nlm.nih.gov/entrez/viewer.fcgi?db=nucleotide&val=22615028) [AR228075](http://www.ncbi.nlm.nih.gov/entrez/viewer.fcgi?db=nucleotide&val=27266821) | [65164](http://www.ncbi.nlm.nih.gov/entrez/query.fcgi?db=gene&cmd=Retrieve&dopt=Graphics&list_uids=65164) |  |
| *Psa* | Aminopeptidase puromycin sensitive | 10q31 | 17q21 | NW-047338 | [BD069403](http://www.ncbi.nlm.nih.gov/entrez/viewer.fcgi?db=nucleotide&val=22615006)  [AR228053](http://www.ncbi.nlm.nih.gov/entrez/viewer.fcgi?db=nucleotide&val=27266799) | [50558](http://www.ncbi.nlm.nih.gov/LocusLink/LocRpt.cgi?l=50558) | HCoV-229E [31] |
| *Psma7* | proteasome (prosome, macropain) subunit, alpha type, 7 | 3q43 | 20q13.33 | XP_342599 | [BD078983](http://www.ncbi.nlm.nih.gov/entrez/viewer.fcgi?db=nucleotide&val=22624586) | [29674](http://www.ncbi.nlm.nih.gov/entrez/query.fcgi?db=gene&cmd=retrieve&dopt=default&list_uids=29674) |  |
| *Pts* | 6-pyruvoyl-tetrahydropterin synthase | 8q23 | 11q22.3-q23.3 | NP_058916 | [BD078942](http://www.ncbi.nlm.nih.gov/entrez/viewer.fcgi?db=nucleotide&val=22624545) | [29498](http://www.ncbi.nlm.nih.gov/entrez/query.fcgi?db=gene&cmd=Retrieve&dopt=Graphics&list_uids=29498) |  |
| *Rfp2* | ret finger protein 2 or Trim13 | 15p12 | 13q14 | NM_005798 | In process | [10206](http://www.ncbi.nlm.nih.gov/entrez/query.fcgi?db=gene&cmd=retrieve&dopt=default&list_uids=10206) |  |
| *Rin2* | ras association (RalGDS/AF-6) domain containing protein JC265; RAB5 interacting protein 2 | 3q41 | 20p11 | XP_230647 | [AR228026](http://www.ncbi.nlm.nih.gov/entrez/viewer.fcgi?db=nucleotide&val=27266772)  [BD069376](http://www.ncbi.nlm.nih.gov/entrez/viewer.fcgi?db=nucleotide&val=22614979) | [311494](http://www.ncbi.nlm.nih.gov/entrez/query.fcgi?db=gene&cmd=Retrieve&dopt=Graphics&list_uids=311494) |  |
| *Ror1* | Receptor tyrosine kinase-like orphan receptor 1 | 5q33 | 1p32-p31 | XP_238402 | [AR228030](http://www.ncbi.nlm.nih.gov/entrez/viewer.fcgi?db=nucleotide&val=27266776)[BD069380](http://www.ncbi.nlm.nih.gov/entrez/viewer.fcgi?db=nucleotide&val=22614983) | [117094](http://www.ncbi.nlm.nih.gov/entrez/query.fcgi?db=gene&cmd=Retrieve&dopt=Graphics&list_uids=117094) |  |
| *Rps18* | S18 ribosomal protein, cytosolic, substrate for Ca2+/ calmodulin-activated protein kinase II | 5q24 | 6p21.3 | XP_232915 | [AR228028](http://www.ncbi.nlm.nih.gov/entrez/viewer.fcgi?db=nucleotide&val=27266774)  [BD069378](http://www.ncbi.nlm.nih.gov/entrez/viewer.fcgi?db=nucleotide&val=22614981) | [298014](http://www.ncbi.nlm.nih.gov/entrez/query.fcgi?db=gene&cmd=Retrieve&dopt=Graphics&list_uids=298014) |  |
| *Rraga* | Ras-related GTP binding A | 5q32 | 9p21.3 | NP_446425 | [BD078977](http://www.ncbi.nlm.nih.gov/entrez/viewer.fcgi?db=nucleotide&val=22624580) | [117044](http://www.ncbi.nlm.nih.gov/entrez/query.fcgi?db=gene&cmd=Retrieve&dopt=Graphics&list_uids=117044) | ADV[32] |
| *Ryk* | Receptor-like tyrosine kinase | 8q32 | 3q22 | XP_343459 | [BD078959](http://www.ncbi.nlm.nih.gov/entrez/viewer.fcgi?db=nucleotide&val=22624562) | [140585](http://www.ncbi.nlm.nih.gov/entrez/query.fcgi?db=gene&cmd=retrieve&dopt=default&list_uids=140585) |  |
| *S100a6/*  *S100a1* | S100 calcium binding protein A6 (calcyclin)/ S-100 protein, alpha chain | 2q34 | 1q21 | NP_445937  XP_215606 | | [AR228013](http://www.ncbi.nlm.nih.gov/entrez/viewer.fcgi?db=nucleotide&val=27266759)  [BD069363](http://www.ncbi.nlm.nih.gov/entrez/viewer.fcgi?db=nucleotide&val=22614966)  [BD079030](http://www.ncbi.nlm.nih.gov/entrez/viewer.fcgi?db=nucleotide&val=22624633)  [BD078981](http://www.ncbi.nlm.nih.gov/entrez/viewer.fcgi?db=nucleotide&val=22624584)  [BD079031](http://www.ncbi.nlm.nih.gov/entrez/viewer.fcgi?db=nucleotide&val=22624634)  [BD079034](http://www.ncbi.nlm.nih.gov/entrez/viewer.fcgi?db=nucleotide&val=22624637)  [BD079029](http://www.ncbi.nlm.nih.gov/entrez/viewer.fcgi?db=nucleotide&val=22624632)  [BD079035](http://www.ncbi.nlm.nih.gov/entrez/viewer.fcgi?db=nucleotide&val=22624638) | | --- | | [85247](http://www.ncbi.nlm.nih.gov/entrez/query.fcgi?db=gene&cmd=Retrieve&dopt=Graphics&list_uids=85247)  [295214](http://www.ncbi.nlm.nih.gov/entrez/query.fcgi?db=gene&cmd=retrieve&dopt=default&list_uids=295214) |  |
| *Scmh1* | sex comb on midleg homolog 1 (Drosophila) | 5q36 | 1p34 | XP_342901 | In process | [22955](http://www.ncbi.nlm.nih.gov/LocusLink/LocRpt.cgi?l=22955) |  |
| *Serp1* | Stress-associated endoplasmic reticulum protein 1 | 15q11 | 3q25.1 | NM_030835 | [BD078962](http://www.ncbi.nlm.nih.gov/entrez/viewer.fcgi?db=nucleotide&val=22624565) | [80881](http://www.ncbi.nlm.nih.gov/entrez/query.fcgi?db=gene&cmd=retrieve&dopt=default&list_uids=80881) |  |
| *Srp19* | signal recognition particle 19kDa | 18p12 | 5q21-q22 | NW_047510 | [BD078963](http://www.ncbi.nlm.nih.gov/entrez/viewer.fcgi?db=nucleotide&val=22624566) | [291685](http://www.ncbi.nlm.nih.gov/entrez/query.fcgi?db=gene&cmd=Retrieve&dopt=Graphics&list_uids=291685) | HCV,  Sinbis,  SFV [33-35] |
| *Stmn1* | Stathmin, microtubule-depolymerizing protein | 8q31 | 1p36.1-p35 | XP_343442 | [AR228025](http://www.ncbi.nlm.nih.gov/entrez/viewer.fcgi?db=nucleotide&val=27266771)  [BD069375](http://www.ncbi.nlm.nih.gov/entrez/viewer.fcgi?db=nucleotide&val=22614978) | [363108](http://www.ncbi.nlm.nih.gov/entrez/query.fcgi?db=gene&cmd=Retrieve&dopt=Graphics&list_uids=363108) |  |
| *Tpm1* | tropomyosin 1 (alpha) | 8q24 | 15q22.1 | NW_047799 | [BD078934](http://www.ncbi.nlm.nih.gov/entrez/viewer.fcgi?db=nucleotide&val=22624537) | [24851](http://www.ncbi.nlm.nih.gov/entrez/query.fcgi?db=gene&cmd=Retrieve&dopt=Graphics&list_uids=24851) |  |
| *Trim52* | Tripartite motif-containing 52 | 15q22 | 5q35.3 | XM_224468 | [BD079011](http://www.ncbi.nlm.nih.gov/entrez/viewer.fcgi?db=nucleotide&val=22624614)  [BD079005](http://www.ncbi.nlm.nih.gov/entrez/viewer.fcgi?db=nucleotide&val=22624608)  [BD079002](http://www.ncbi.nlm.nih.gov/entrez/viewer.fcgi?db=nucleotide&val=22624605) | [290458](http://www.ncbi.nlm.nih.gov/LocusLink/LocRpt.cgi?l=290458) |  |
| *Tsec-2^+^/*  Mthfd1^-^ | tsec-2^+^/C1-tetrahydrofolate synthase^-^ | 6q24 | 14q24 | NW_047761  NM_022508 | [AR228055](http://www.ncbi.nlm.nih.gov/entrez/viewer.fcgi?db=nucleotide&val=27266801)  [BD069405](http://www.ncbi.nlm.nih.gov/entrez/viewer.fcgi?db=nucleotide&val=22615008) | [299152](http://www.ncbi.nlm.nih.gov/entrez/query.fcgi?db=gene&cmd=Retrieve&dopt=Graphics&list_uids=299152)  [64300](http://www.ncbi.nlm.nih.gov/entrez/query.fcgi?db=gene&cmd=Retrieve&dopt=Graphics&list_uids=64300) |  |
| *Ube1c* | ubiquitin-activating enzyme E1C | 4q34 | 3p24.3-p13 | NP_476553 | [AR228029](http://www.ncbi.nlm.nih.gov/entrez/viewer.fcgi?db=nucleotide&val=27266775)  [BD069379](http://www.ncbi.nlm.nih.gov/entrez/viewer.fcgi?db=nucleotide&val=22614982) | [117553](http://www.ncbi.nlm.nih.gov/entrez/query.fcgi?db=gene&cmd=Retrieve&dopt=Graphics&list_uids=117553) |  |
| *Zfp207* | zinc finger protein 207 | 10q26 | 17q12 | XP_221231 | [BD078995](http://www.ncbi.nlm.nih.gov/entrez/viewer.fcgi?db=nucleotide&val=22624598) | [303763](http://www.ncbi.nlm.nih.gov/entrez/query.fcgi?db=gene&cmd=Retrieve&dopt=Graphics&list_uids=303763) |  |
| *Znf7* | Zinc finger protein 7 | 7q34 | 8q24 | XP_235457 | In process | [315101](http://www.ncbi.nlm.nih.gov/entrez/query.fcgi?db=gene&cmd=Retrieve&dopt=Graphics&list_uids=315101) |  |

References
